# SECONDHAND SMOKE: Parental Smoking May Set Up Children for Atherosclerosis

**DOI:** 10.1289/ehp.118-a200a

**Published:** 2010-05

**Authors:** Adrian Burton

**Affiliations:** **Adrian Burton** is a biologist living in Spain who also writes regularly for *The Lancet Oncology*, *The Lancet Neurology*, and *Frontiers in Ecology and the Environment.*

Children frequently exposed to secondhand tobacco smoke may be at greater risk of developing atherosclerosis as adults, suggests new research published in the March 2010 issue of *Circulation: Cardiovascular Quality and Outcomes*. “Passive smoking has been associated with an increased risk of atherosclerosis in adults by altering arterial structure and lipid profiles, but there is growing evidence the trouble might begin in childhood,” explains first author Katariina Kallio, a research fellow at the Research Centre of Applied and Preventive Cardiovascular Medicine, University of Turku, Finland. “Our study shows changes do occur in the intima-media thickness of the arteries of healthy adolescents exposed to smoke, confirming this fear.”

The study, which involved 494 healthy 13-year-olds, measured three preclinical indicators of atherosclerosis: the carotid and aortic intima-media thickness (i.e., the thickness of the walls of these arteries), flow-mediated dilation of the brachial artery (a measure of endothelial function), and plasma apolipoprotein B (ApoB) levels (a measure of circulating atherogenic lipoproteins).

The researchers divided the children into low-, intermediate-, and high-exposure groups, which were determined on the basis of each child’s average serum cotinine level (0.1–0.4 ng/mL, 0.41–0.7 ng/mL, and 0.71–4.1 ng/mL, respectively) as measured at different times since age 8 years. High-resolution ultrasound scans showed the intima-media of the carotid artery was thicker in children in the high- and intermediate-exposure groups than in the low-exposure group. The same was true of the aortic intima-media. In addition, flow-mediated dilation in the brachial artery was significantly reduced in the high-exposure group.

These preclinical signs of atherosclerosis were accompanied by significantly increased ApoB levels in the high-exposure group compared with the low-exposure group. Their ApoB/ApoA-1 ratios also were significantly higher, a predictor of atherosclerosis and endothelial dysfunction in adults. Associations persisted after accounting for other atherosclerosis risk factors including serum lipids, sex, pubertal status, diastolic blood pressure, and body mass index.

“Intima-media thickness may be increased by a direct toxic effect of tobacco smoke, the enhanced binding of platelets to vessels causing the growth of vascular smooth muscle, or perhaps lipid peroxidation; we are not sure,” says Kallio. “Nor are we sure why ApoB is increased, although we believe liver enzyme function might be involved.”

“Whether the changes seen are reversible on removing children from smoke exposure, and how long this would take, are also currently unknown,” comments John Cockcroft of the Wales Heart Research Institute, Cardiff University School of Medicine, who was not involved in the study.

The publication of these findings coincides with a 24 March 2010 call by the Royal College of Physicians (RCP) to ban smoking in cars in the United Kingdom to reduce children’s exposure to secondhand smoke. Several U.S. states and parts of Australia and Canada already ban smoking specifically in cars carrying children. “What is clear,” says Cockcroft, “is that [this Finnish study] provides further evidence to support the RCP’s call for a ban on exposing children to smoke inhalation—a proposal that the authors themselves support.”

